# Indocyanine Green and Fluorescein Videoangiography for the Assessment of Collateral Circulation in Posterior Circulation Aneurysm Clipping: A Case Report and Review

**DOI:** 10.2174/0115734056256001250812075213

**Published:** 2025-08-22

**Authors:** Alejandro Serrano-Rubio, Carlos-Fernando Nicolas-Cruz, Sharon Trujillo, Brenda-Susana Hernández-Barrera, Ambar-Elizabeth Riley-Moguel, Julian-Moises Enriquez-Alvarez, Ana-Margarita Martinez-Caceres, Rafael Sánchez-Mata, Daniel Figueroa-Zelaya, Ernesto Roldan-Valadez, Edgar Nathal

**Affiliations:** 1 Department of Vascular Neurosurgery, National Institute of Neurology and Neurosurgery “Manuel Velasco Suárez”, 14267, Mexico City, Mexico; 2 Division of Research, Instituto Nacional de Rehabilitación “Luis Guillermo Ibarra Ibarra”, 14389, Mexico City, Mexico; 3 I.M. Sechenov First Moscow State Medical University (Sechenov University), Radiology Department, 119992, Moscow, Russia

**Keywords:** Indocyanine green, Fluorescein video angiography, Posterior circulation aneurysm, Microsurgical clipping, Collateral circulation, Intraoperative imaging

## Abstract

**Background:**

Microsurgical treatment of posterior circulation aneurysms remains challenging due to their deep location, complex anatomical exposure, and close proximity to critical neurovascular structures. Ensuring adequate collateral circulation is paramount for preventing ischemic complications. Indocyanine Green (ICG) and Fluorescein Video Angiography (FL-VAG) have emerged as effective intraoperative tools for assessing cerebral perfusion and guiding surgical decision-making.

**Case Presentation:**

We report the case of a 29-year-old male presenting with a thunderclap headache, nausea, and vomiting, subsequently diagnosed with a fusiform aneurysm at the P2-P3 junction of the left posterior cerebral artery. The patient underwent a subtemporal approach with partial posterior petrosectomy for aneurysm clipping and remodeling. Initially, an STA-P3 and PITA-P3 bypass were considered; however, intraoperative ICG and FL-VAG confirmed sufficient retrograde collateral flow, allowing the bypass procedure to be avoided. Postoperative imaging demonstrated patent circulation in the occipitotemporal region without ischemic compromise.

**Conclusion:**

This case highlights the crucial role of intraoperative fluorescence imaging in refining surgical strategies for complex aneurysm clipping. ICG and FL-VAG enhance surgical precision by providing real-time perfusion assessment, reducing the need for additional vascular interventions, and improving patient outcomes.

## 1 INTRODUCTION

Intracranial aneurysms, particularly those located in the posterior circulation, present significant surgical challenges due to their deep anatomical location, complex morphology, and association with critical neurovascular structures [1]. Advances in microsurgical techniques have improved patient outcomes; however, ensuring adequate intraoperative vascular assessment remains crucial for preventing ischemic complications and optimizing surgical strategies [2].

The fusiform aneurysms of the posterior cerebral artery are extremely rare, representing less than 0.1% of the cerebral aneurysms [3]. They usually arise on the P1 segment in 26%, at the P1P2 junction in 16%, 45% on P2, and 13% on a further distal segment. Fusiform aneurysms of the posterior cerebral arteries are even less often, slightly less common in the P2 and extremely rare in the P3 segment [3]. The subtemporal approach with or without resection of the temporal lobe is preferred by some authors, but there is a high risk for the basal temporal veins being injured, and this approach requires too much retraction of the temporal lobe. Despite the rich collateral circulation in the posterior cerebral artery (PCA), complications such as infarction in the uncus and part of the inferior posterior temporal lobe and hemianopsia, because of occipital lobe infarction, can occur. Due to these problems, some authors suggest PCA revascularization, and some others recommend a combination of microvascular by-pass and endovascular parent occlusion [4].

Indocyanine Green (ICG) and Fluorescein Video Angiography (FL-VAG) have emerged as powerful intraoperative tools that enable real-time visualization of cerebral perfusion, vessel patency, and bypass graft functionality [5, 6]. ICG video angiography, introduced as an alternative to intraoperative digital subtraction angiography (DSA), provides high-resolution visualization of the arterial and venous phases without the need for contrast injection via an arterial catheter [7]. Meanwhile, FL-VAG enhances visualization of perforators and microvascular anastomoses, proving particularly beneficial in complex cerebrovascular procedures [8].

In cases involving fusiform or dysplastic aneurysms, which often lack a well-defined neck for direct clipping, bypass surgery may be required to maintain adequate distal perfusion [9]. The decision to proceed with bypass reconstruction or direct clipping is heavily dependent on intraoperative evaluation of collateral circulation [10]. This case report illustrates the critical role of ICG and FL-VAG in guiding surgical decision-making for a complex fusiform aneurysm of the PCA.

By integrating multimodal intraoperative imaging, neurosurgeons can make real-time modifications to their surgical approach, potentially avoiding unnecessary bypass procedures while ensuring optimal cerebral perfusion.

A preoperative assessment of aneurysm wall characteristics can contribute to the decision-making for the treatment of unruptured cerebral aneurysms. A 4D CTA can provide the dynamics of an aneurysm wall relating to aneurysm growth, bleb formation, and rupture point. There are studies that capture the dynamic nuances of an aneurysm using deep learning-based supervised machine learning for point-level risk assessment in cerebral aneurysms using 4D-CTA. These types of studies, which utilize artificial intelligence techniques such as deep learning, can guide future research that combines intraoperative fluorescence methods with quantitative results [11]. The use of ICG video angiography and FL-VAG has similar rates of clip repositioning and parent vessel stenosis when compared with either intraoperative or postoperative DSA [12, 13]. The routine use of ICG video angiography and FL-VAG compared to routine DSA led to a significant cost reduction based on billing for operating room time and catheter angiography itself [14]. The following case highlights how ICG and FL-VAG can refine intraoperative judgment, enhance surgical precision, and improve postoperative outcomes in the management of complex posterior circulation aneurysms.

## 2 CASE PRESENTATION

A 29-year-old male with a history of hypertension and smoking presented to the emergency department with a sudden-onset thunderclap headache, accompanied by nausea and vomiting. On initial neurological examination, the patient was alert and reactive, with no cranial nerve dysfunction or focal neurological deficits, except for nuchal rigidity. Based on clinical assessment, he was classified as Hunt & Hess grade I.

### 2.1 Initial Imaging and Diagnosis

**Fig. (1):**
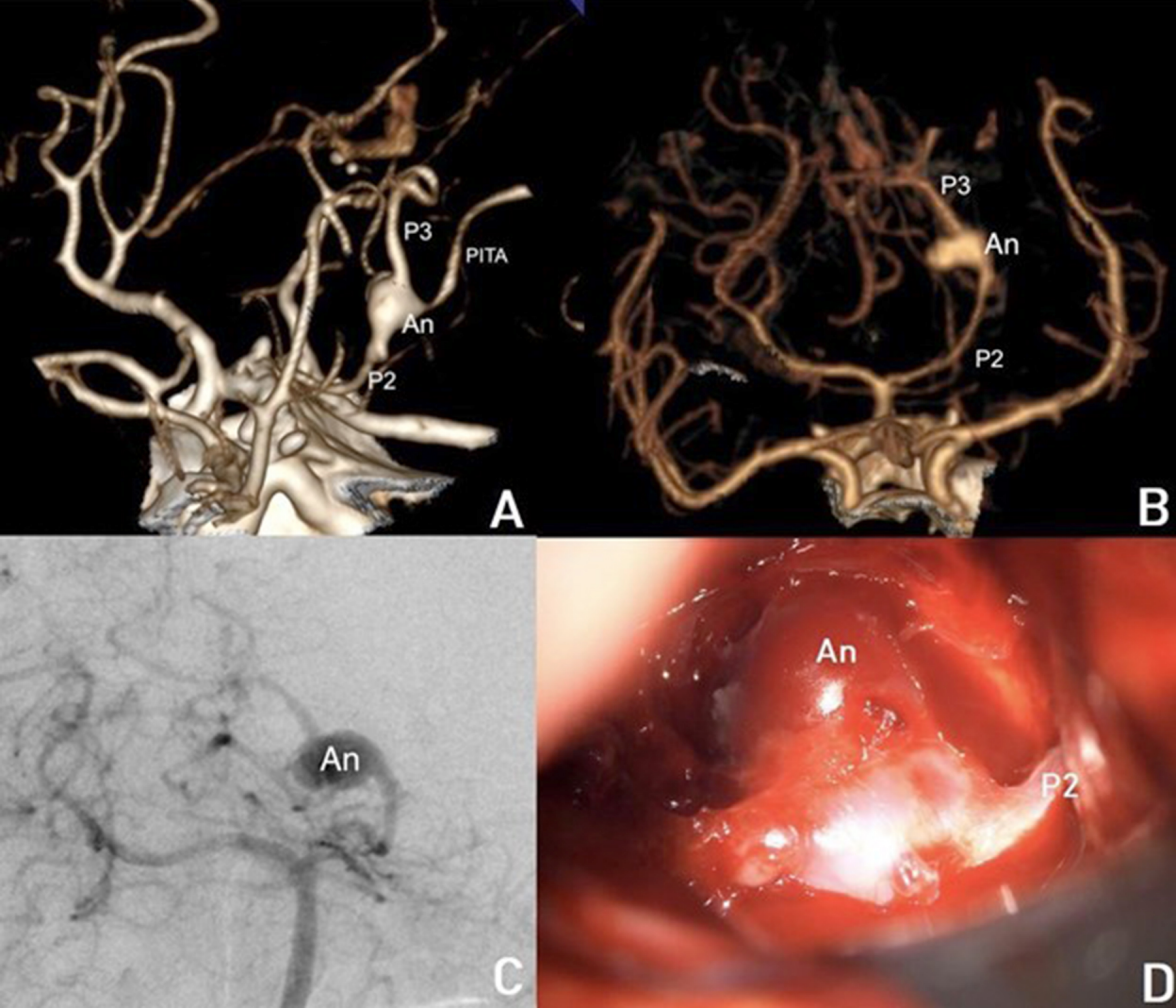
(**A**) CT angiography (CTA), sagittal reconstruction, demonstrating a left P2-P3 aneurysm at the origin of the posteroinferior temporal artery (PITA) showing a diameter of 6.88 mm and 8.75 of height. (**B**) CTA, coronal reconstruction, depicting the aneurysm neck and dome projection at the P2-P3 junction, this image reflects a fusiform aneurysm and a bilobulated pattern. (**C**) Digital subtraction angiography (DSA), showing an oblique projection of a selective injection in the left vertebral artery, showing the relationship between the aneurysm and perforator branches, and the aneurysm and its characteristics and dimensions previously mentioned (**D**). Intraoperative surgical view, following arachnoid dissection, exposing the aneurysm and surrounding neurovascular structures before clipping.

A non-contrast cranial computed tomography (CT) scan revealed a Fisher IV subarachnoid hemorrhage (SAH) without signs of hydrocephalus (Fig. **1A**) highlighting the characteristics of a left P2-P3 aneurysm). To further evaluate the etiology, a cranial CT angiography (CTA) was performed, identifying a complex saccular aneurysm at the P2-P3 junction of the left posterior cerebral artery (PCA) (Fig. **1B**), a fusiform aneurysm and a bilobulated pattern.). Digital subtraction angiography (DSA) provided further characterization, demonstrating a fusiform aneurysm at the branching point of the posterior inferior temporal artery (PITA) in the P2-P3 segment of the PCA (Fig. **1C**), presenting the relationship between the aneurysm and perforator branches).

### 2.2 Surgical Approach and Intraoperative Evaluation

The patient underwent a left subtemporal approach with partial posterior petrosectomy, following a question mark incision technique, as described by Uda *et al*. [15]. The initial surgical plan included:

(1) Superficial temporal artery to P3 (STA-P3) end-to-side bypass.

(2) Posterior inferior temporal artery to P3 (PITA-P3) end-to-side transposition bypass.

However, intraoperative evaluation using multimodal fluorescence angiography altered the surgical strategy as shown in Fig. (**1D**). Indocyanine green videoangiography (ICG-VA) with Flow 800 analysis demonstrated retrograde blood flow from the PITA, thereby negating the need for a PITA-P3 transposition bypass. The aneurysm was secured using a 9 mm, 90° angle fenestrated permanent clip, which redirected flow toward the posterior temporal artery, and an additional 3 mm straight mini-clip was placed to isolate the P3 segment as shown in Fig. (**2A**).

**Fig. (2):**
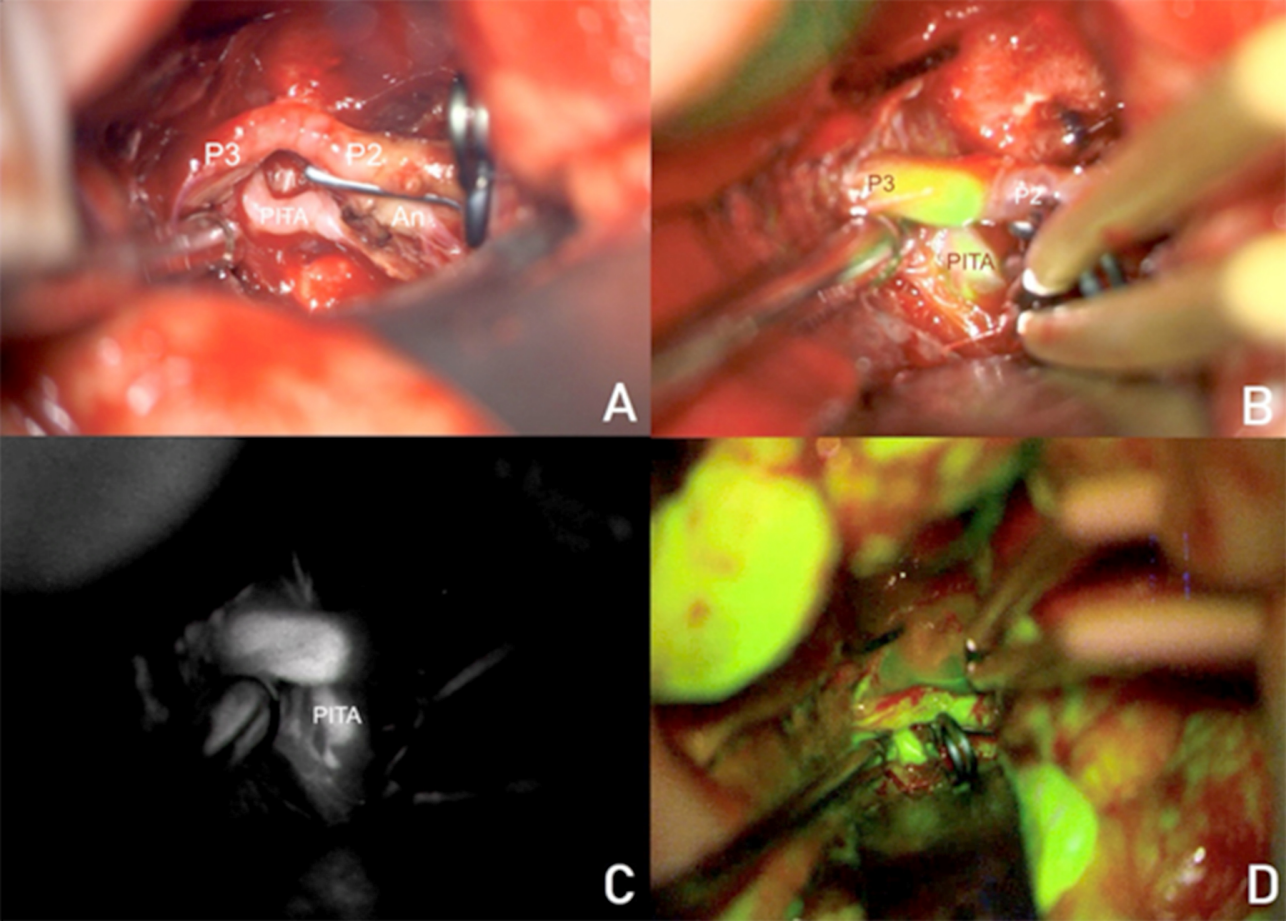
(**A**) Intraoperative view of aneurysm clipping and remodeling, using a 9 mm, 90° angle fenestrated permanent clip, along with a 3 mm straight miniclip for segmental occlusion. (**B**) Intraoperative indocyanine green videoangiography (ICG-VA) demonstrates collateral flow from the P3 segment to the PITA and distal posterior cerebral artery (PCA), supporting the decision to avoid bypass. (**C**) Video angiography showing the perfusion flow of the P3 segment. (**D**) Intraoperative fluorescein videoangiography (FL-VAG), confirming preserved perfusion in adjacent brain regions, with no evidence of significant hypoperfusion, attributed to perforator collaterality.

Additionally, fluorescein videoangiography (FL-VAG) was employed to assess cerebral perfusion and identify areas at risk for ischemia (Fig. **2B**). These assessments confirmed sufficient collateral circulation as shown in Fig. (**2C**), allowing the surgical team to proceed with direct aneurysm clipping and remodeling (Fig. **2D**).

### 2.3 Postoperative Course and Outcome

The patient emerged from anesthesia alert, exhibiting a mild third cranial nerve (oculomotor) palsy but without motor or sensory deficits possibly caused by manipulation during surgery or by direct compression of the nerve by the aneurysm; this palsy is an entity that typically occurs suddenly in conjunction with subarachnoid [16]. The recovery of third nerve function after surgery is well-documented, typically beginning within the first month after treatment and taking several months. Ptosis is the first symptom to appear [17].

**Fig. (3):**
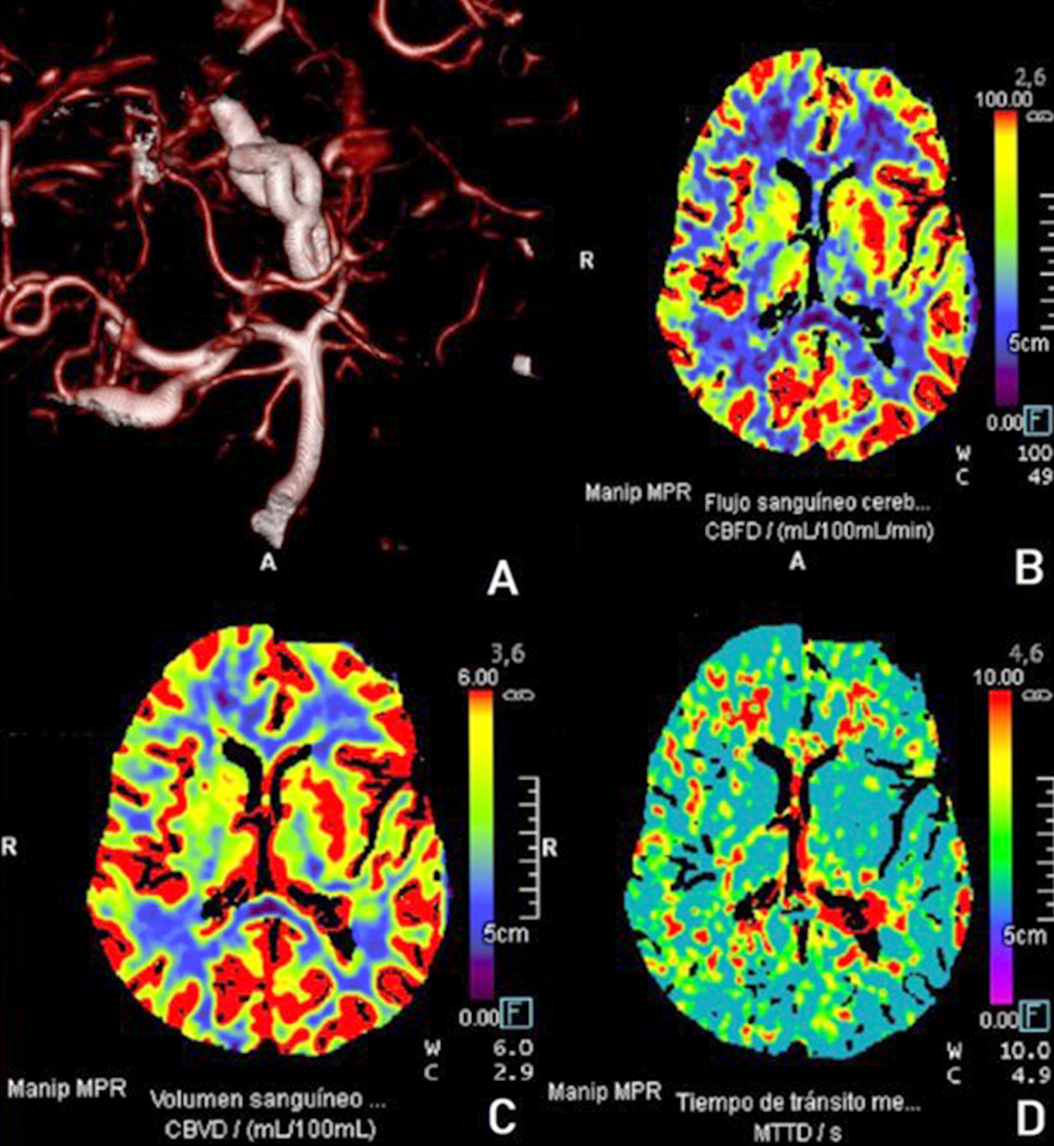
(**A**) Postoperative CTA, oblique reconstruction, demonstrating aneurysm clipping configuration, with preserved distal flow in the P3 segment and PITA. (**B**) Cerebral blood flow (CBF) map, confirming adequate perfusion postoperatively. (**C**) Cerebral blood volume (CBV) assessment, showing preserved hemodynamics. (**D**) Mean transit time (MTT) imaging demonstrates satisfactory postoperative cerebral perfusion and the absence of ischemic compromise.

Postoperative CT angiography, as shown in Fig. (**3A**), and perfusion CT, as shown in Fig. (**3B**) confirmed preserved vascular patency in the occipitotemporal region (Fig. **3C**), with no areas of hypoperfusion or infarction ( Fig. **3D**).

The patient remained under neurological surveillance for 2 days and was subsequently discharged on postoperative day 4 with a modified Rankin Scale (mRS) score of 1, indicating mild symptoms but no significant disability.

## 3 DISCUSSION

Although posterior circulation aneurysms account for approximately 15% of all intracranial aneurysms, they pose a higher risk of rupture and worse clinical outcomes compared to their anterior circulation counterparts [18]. Despite advancements in endovascular therapy, microsurgical treatment remains superior in the long term, demonstrating lower rates of rebleeding, lower recurrence rates, and higher aneurysm obliteration rates [19, 20]. However, the treatment of wide-neck aneurysms, giant thrombotic aneurysms, or aneurysms involving critical branching and perforating arteries presents challenges for both endovascular and microsurgical approaches [10].

### 3.1 Role of Intraoperative Angiography in Aneurysm Surgery

Intraoperative adjunct techniques are essential for evaluating aneurysm remnants, assessing the patency of parent and branching arteries, and identifying potential bypass grafts during surgery. Since the 1940s and 1960s, Fluorescein (FL) and Indocyanine Green (ICG) Video Angiography (VAG) have been widely used across multiple surgical specialties, including cardiovascular, ophthalmologic, and gastrointestinal surgery, respectively [7, 21]. These techniques provide real-time assessment of cerebral blood flow, enabling the detection of vascular compromise during temporary occlusion in complex lesions. Studies have reported a concordance rate of 90-100% in detecting aneurysmal remnants and parent or branch vessel involvement using ICG and FL-VAG [22-24].

Traditional intraoperative digital subtraction angiography (DSA) remains the gold standard for evaluating cerebral blood flow and ischemic risk during aneurysm surgery. However, DSA requires a hybrid operating room, which may not always be available [8, 25]. As an alternative, ICG-VA and FL-VAG offer immediate, high-resolution vascular mapping, eliminating the need for intraoperative catheter-based contrast injections.

FLOW 800 (Zeiss Meditec, Germany) is a software program that allows ICG data to be analyzed in a semiquantitative manner through a color delay map, which identifies the direction and sequence of blood flow relative to the surrounding vasculature via fluorescence dynamics [26]. The time when a fluorescence signal appears is recorded for each image point and visualized in a color-encoded map (Fig. **3B**). An intuitive scale mark for the early appearance of fluorescence in red and the late appearance in blue. Therefore, red represents the initial blood inflow, and the other color gradients show the subsequent sequences of flow. The displayed intensity values and seconds (SI units) are rounded values [26].

The FLOW 800 data have the potential to provide information only on differences in superficial hemodynamic flow in cortical vessels [26] (Fig. **3C**-**D**). The intraoperative Doppler can aid in obtaining a quantitative measure of the flow in the aneurysm and collateral circulation. Therefore, further research would be desirable to find a method for measuring the absolute rate of flow and collateral flow in aneurysm surgery.

### 3.2 ICG *vs*. Fluorescein Video Angiography: Advantages and Limitations

Due to the deep anatomical location of posterior circulation aneurysms, it is crucial to understand the benefits and limitations of these fluorescence-based intraoperative imaging techniques [27] (Table **1**). ICG-VA requires an infrared camera and digital overlay technology, which can be viewed via a separate display or injected directly into the microscope eyepieces. However, one of its primary advantages is the enhanced visualization of thick-walled vessels, making it particularly useful for evaluating proximal branches and larger perforators [28].

In contrast, FL-VAG offers improved visualization of small vessels, which can be observed directly through the microscope's eyepieces or external displays in real-time. This feature allows for the early detection of brain territories at risk of ischemia, providing immediate feedback on perfusion adequacy [5, 29]. The standard dosage for ICG-VA is a 25 mg bolus, while FL-VAG requires 0.4 mL of a 10% fluorescein solution, both of which are followed by a 20 mL saline flush via intravenous administration. The complication rate for both techniques remains low, at less than 0.2% [6, 30]. ICG side effects are rare: minor adverse reactions (nausea, skin rash) were observed in 0.2% of cases, while hypotension, arrhythmia and anaphylactic shock were observed in 0.05% of cases [16]. The adverse effects of FL-VAG are rare and may bemild, including temporary skin, mucosa, and urine staining in the first 24-48h after surgery, but the phenomenon seems self-limited and without sequelae. In rare cases (1:1900), more severe complications can be observed, such as cardiac effects, respiratory reactions, or seizures, and they seem to be related to the use of higher doses and intrathecal injections [16].

Despite their advantages, one of the primary limitations of these techniques is their qualitative nature. While they provide visual confirmation of collateral flow, they do not quantify whether the detected flow is sufficient to prevent ischemia [13, 31].

Therefore, intraoperative monitoring techniques such as motor and somatosensory evoked potentials (MEPs/SSEPs) play a complementary role in assessing neural function and ischemic risk. These modalities also facilitate real-time communication between the surgical team, anesthesiologists, and electrophysiologists, ensuring optimal patient outcomes following aneurysm surgery [32].

## 4 STUDY LIMITATIONS

This case report uses ICG-VA and FL-VA to provide real-time intraoperative visualization and demonstrate collateral flow from the P3 segment to the PITA and distal posterior cerebral artery (PCA). However, a quantitative method for measuring flow, such as intraoperative Doppler, could be useful to make the results more reproducible and draw ﻿definite conclusions about its superiority over other techniques.

## CONCLUSION

The present case highlights the critical role of intraoperative fluorescence angiography in the microsurgical treatment of posterior circulation aneurysms. Despite advancements in endovascular techniques, microsurgical clipping remains the preferred approach for complex aneurysms involving branching or perforating arteries, where durability and complete obliteration are key considerations. The combination of indocyanine green videoangiography (ICG-VA) and fluorescein videoangiography (FL-VAG) provided real-time intraoperative visualization, enabling precise surgical decision-making and eliminating the need for unnecessary bypass procedures.

This case reinforces several key principles in aneurysm surgery:

1. Tailored microsurgical strategies remain essential for complex aneurysms, particularly in the posterior circulation.
2. ICG-VA and FL-VAG serve as reliable intraoperative tools, providing high-resolution vascular mapping without the logistical challenges associated with intraoperative digital subtraction angiography (DSA).
3. The qualitative nature of fluorescence angiography necessitates complementary techniques, such as intraoperative electrophysiological monitoring, to ensure optimal neurovascular outcomes.

The integration of advanced intraoperative imaging modalities continues to enhance surgical precision, reduce morbidity, and improve patient outcomes in cerebrovascular surgery. As fluorescence angiography techniques evolve, further research is warranted to standardize protocols, optimize dosing strategies, and refine their predictive value in preventing ischemic complications.

Ultimately, this case underscores the importance of a patient-specific, imaging-driven approach in the management of complex intracranial aneurysms, ensuring both vascular preservation and long-term treatment efficacy.

## DECLARATION OF GENERATIVE AI AND AI-ASSISTED TECHNOLOGIES IN THE WRITING PROCESS

During the preparation of this work, the author(s) used approved software like Grammarly to correct grammar and style. After using this tool/service, the author(s) reviewed and edited the content as needed and took full responsibility for the content of the publication.

## AUTHORS' CONTRIBUTIONS

A.S.-R: Data acquisition, data analysis and interpretation, research, writing original draft, review and editing of manuscript, design and development of tables and figures; C.F.N.C.: Data acquisition, interpretation of data analysis, research, writing original draft, review and editing of the manuscript, supervision; S.T.: Data analysis and interpretation, research, review and editing of manuscript, design and development of figures; J.M.E.Á: Data analysis and interpretation, research, review and editing of manuscript, design and development of figures; B.S.H.-B.: Writing original draft, research, review, and editing of manuscript; A.E.R.M.: Data analysis and interpretation, research, review, and editing of manuscript, design and development of figures; A.M.M.C: Data analysis and interpretation, research, review and editing of manuscript, design and development of figures; R.S.-M: Data analysis and interpretation, research, design and development of figures. D.F.-Z.: Data analysis and interpretation, research, design and development of figures. E.N.: Data analysis and interpretation, research, review and editing of manuscript, design and development of tables and figures, supervision; E.R.-V.: Data analysis and interpretation, research, review and editing of manuscript, design and development of tables and figures, supervision.

## Figures and Tables

**Table 1:** Comparative Characteristics of Indocyanine Green and Fluorescein Video Angiography. This table summarizes the main differences between Indocyanine Green (ICG) and Fluorescein video angiography techniques, focusing on physical properties, imaging performance, and clinical utility during intraoperative vascular assessment.

**Characteristics**	**Fluorescein**	**Indocyanine Green (ICG)**
Target vessels	Optimal for small perforating vessels	Optimal for thick-walled or larger vessels.
Molecular weight	376 Da	775 Da
Filter examples	Yellow 560	Flow 800
Imaging output	Qualitative	Qualitative / Semi-quantitative.
Standard dose	0.4 mL of 10% solution	25 mg intravenous bolus.
Optimal visualization site	Deep surgical field	Superficial surgical field.
Main advantages	Low cost	Allows rapid and repeat assessments.
Primary limitations	Inefficient for repeated dosing	Higher cost; requires compatible microscope filters.

## Data Availability

All data generated or analyzed during this study are included in this published article.

## LIST OF ABBREVIATIONS

CBF: Cerebral blood flow
CBV: Cerebral blood volume
CTA: Computed tomography angiography
DSA: Digital subtraction angiography
FL-VAG: Fluorescein videoangiography
ICG-VA: Indocyanine green videoangiography
MTT: Mean transit time
PCA: Posterior cerebral artery
PITA: Posteroinferior temporal artery

## STANDARDS OF REPORTING

CARE guidelines were followed.
